# Conditional deletion of *Ndufs4* in dopaminergic neurons promotes Parkinson’s disease-like non-motor symptoms without loss of dopamine neurons

**DOI:** 10.1038/srep44989

**Published:** 2017-03-22

**Authors:** Won-Seok Choi, Hyung-Wook Kim, François Tronche, Richard D. Palmiter, Daniel R. Storm, Zhengui Xia

**Affiliations:** 1 Department of Environmental and Occupational Health Sciences, University of Washington, Seattle, WA 98195, USA; 2 School of Biological Sciences and Technology, College of Natural Sciences, College of Medicine, Chonnam National University, Gwangju 61186, Korea; 3 College of Life Sciences, Sejong University, Seoul 05006, Korea; 4 Sorbonne Universités, Université Pierre et Marie Curie, UMR_CR18, Neuroscience, Paris-Seine, F-75005, Paris; 5 Howard Hughes Medical Institute and Department of Biochemistry, University of Washington, Seattle, WA 98195, USA; 6 Department of Pharmacology, University of Washington, Seattle, WA 98195, USA

## Abstract

Reduction of mitochondrial complex I activity is one of the major hypotheses for dopaminergic neuron death in Parkinson’s disease. However, reduction of complex I activity in all cells or selectively in dopaminergic neurons via conditional deletion of the *Ndufs4* gene, a subunit of the mitochondrial complex I, does not cause dopaminergic neuron death or motor impairment. Here, we investigated the effect of reduced complex I activity on non-motor symptoms associated with Parkinson’s disease using conditional knockout (cKO) mice in which *Ndufs4* was selectively deleted in dopaminergic neurons (*Ndufs4* cKO). This conditional deletion of *Ndufs4,* which reduces complex I activity in dopamine neurons, did not cause a significant loss of dopaminergic neurons in substantia nigra pars compacta (SNpc), and there was no loss of dopaminergic neurites in striatum or amygdala. However, *Ndufs4* cKO mice had a reduced amount of dopamine in the brain compared to control mice. Furthermore, even though motor behavior were not affected, *Ndufs4* cKO mice showed non-motor symptoms experienced by many Parkinson’s disease patients including impaired cognitive function and increased anxiety-like behavior. These data suggest that mitochondrial complex I dysfunction in dopaminergic neurons promotes non-motor symptoms of Parkinson’s disease and reduces dopamine content in the absence of dopamine neuron loss.

Parkinson’s disease is the second most common progressive neurodegenerative disorder. It is characterized by the loss of dopaminergic neurons in the substantia nigra pars compacta (SNpc) of the brain and by motor dysfunction including bradykinesia, rigidity, and tremor^1^,^2^,^3^. Although the mechanisms of dopaminergic neuron death in Parkinson’s disease are not completely established, inhibition of mitochondrial complex I has been a leading hypothesis^4^. This hypothesis was originally based on the observation that accidental exposure to 1-methyl-4-phenyl-1,2,3,6-tetrahydropyridine (MPTP) caused Parkinsonism in humans, as well as the discovery that a metabolite of MPTP, MPP^+^, is a mitochondrial complex I inhibitor^5^,^6^. The complex I inhibition hypothesis was further supported by the finding of reduced complex I activity in various tissues from Parkinson’s disease patients, including familial forms of Parkinson’s disease with the loss-of-function mutation of PTEN-induced putative kinase 1 (PINK1)^7^,^8^,^9^,^10^,^11^,^12^,^13^,^14^. Furthermore, treatment of rodents with MPTP or rotenone, another complex I inhibitor, induces key features of Parkinson’s disease^15^,^16^,^17^,^18^,^19^,^20^,^21^,^22^,^23^,^24^,^25^.

We tested the mitochondrial complex I hypothesis using mouse strains in which the *Ndufs4* gene was deleted. *Ndufs4* encodes an 18 kDa protein that is one of 46 subunits of the mitochondrial complex I; it is required for the complete assembly and function of complex I^26^,^27^,^28^,^29^,^30^,^31^. Systemic deletion of the *Ndufs4* gene starting from embryonic development abolishes complex I activity but does not cause dopaminergic neuron death in culture or in young mice^32^,^33^. Furthermore, inducible deletion of the *Ndufs4* gene in all the cells of adult mice reduces complex I activity by 71% but does not cause dopamine neuron death^34^. We also generated conditional *Ndufs4* knockout mice (cKO) in which *Ndufs4* is specifically deleted in dopaminergic neurons. Complex I activity was reduced by 84% in dopaminergic synaptosomes isolated from the striatum of the *Ndufs4* cKO mice, but these mice do not show dopamine neuron loss, motor deficits, or altered sensitivity to MPTP over a 24-month life-span^34^. These results suggest that reduced mitochondrial complex I activity alone does not cause dopaminergic neuron death during aging nor does it contribute to dopamine neuron toxicity in the MPTP model of Parkinson’s disease.

In addition to motor impairments, many Parkinson’s disease patients also suffer from non-motor symptoms, including anxiety, depression, as well as impairment of olfaction and cognition^35^,^36^,^37^,^38^. These non-motor symptoms may start in the early stages of the Parkinson’s disease prior to the onset of motor symptoms^39^,^40^,^41^. Though the degeneration of serotonergic and noradrenergic neurons may underlie these non-motor symptoms, dopamine transporter-deficient mice exhibit hyperactivity and anxiety responses, suggesting that dysfunction of the dopamine pathway may also contribute^42^,^43^,^44^,^45^,^46^. In this study, we used *Ndufs4* cKO mice to investigate the effects of complex I dysfunction on non-motor symptoms associated with Parkinson’s disease^34^.

## Results

### Loss of *Ndufs4* selectively in dopaminergic neurons does not cause loss of dopaminergic neuron cell bodies or nerve terminals in the substantia nigra

**Figure 1:**
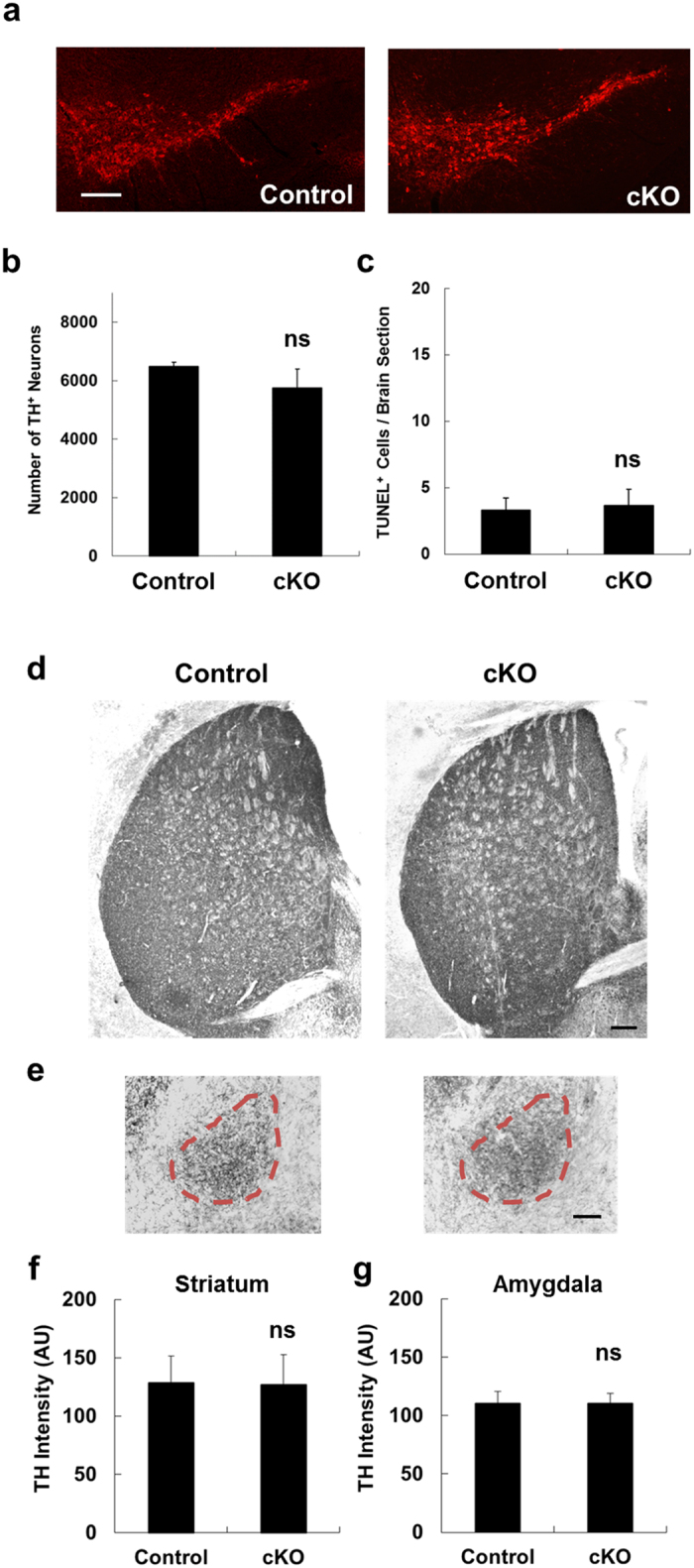
Dopaminergic neuron-specific *Ndufs4* knockout does not cause dopamine neuron death. (**a**) Representative photomicrographs of TH immunostaining of SNpc. cKO: conditional knockout of *Ndufs4* (Dat-cre/*Ndufs4*^lox/lox^) mice; control: *Ndufs4*^lox/lox^ littermates. Bar, 200 μm. (**b**) Quantification of the total number of TH^+^ neurons in the SNpc. (**c**) Quantification of the total number of apoptotic, TUNEL^+^ cells in the SNpc. n.s. not statistically significant. (**e,f**) Representative TH staining of striatal (**e**) and amygdalar (**f**) tissue from Dat-cre/*Ndufs4*^lox/lox^ (cKO) mice and *Ndufs4*^lox/lox^ (Control) mice. Bar, 200 μm. (**g,h**) Quantification of TH staining intensity in the striatum (**g**) and amygdala (**h**). AU, arbitrary units. n.s. not statistically significant.

We previously generated mice in which the *Ndufs4* gene was selectively inactivated in dopaminergic neurons (*Ndufs4* cKO) to achieve selective reduction of mitochondrial complex I activity in dopaminergic neurons^34^. We showed that this conditional *Ndufs4* deletion impaired the activity of mitochondrial complex I in dopaminergic synaptosomes of *Ndufs4* cKO mice^34^. We report here that the number of tyrosine hydroxylase (TH)-positive, dopaminergic neurons is not significantly different in the SNpc of control vs. *Ndufs4* cKO mice (Fig. 1a,b), confirming our previous findings^34^. Furthermore, there was no increase in basal levels of terminal deoxynucleotidyl transferase (TdT)-mediated dUTP nick end labeling (TUNEL, Fig. 1c) in TH+ dopaminergic neurons. Additionally, there was no significant difference in the distribution or intensity of TH immunostaining in the striatum or amygdala, target areas of nigrostriatal or mesolimbic pathways of dopamine neurons in the substantia nigra, respectively (Fig. 1d–g). Together with our previous reports^32^,^33^,^34^, these data suggest that mitochondrial complex I dysfunction does not induce the loss of the cell body or nerve terminals of dopaminergic neurons of substantial nigra.

### *Ndufs4* cKO mice have reduced dopamine in the striatum and amygdala but the level of serotonin is not affected

**Figure 2:**
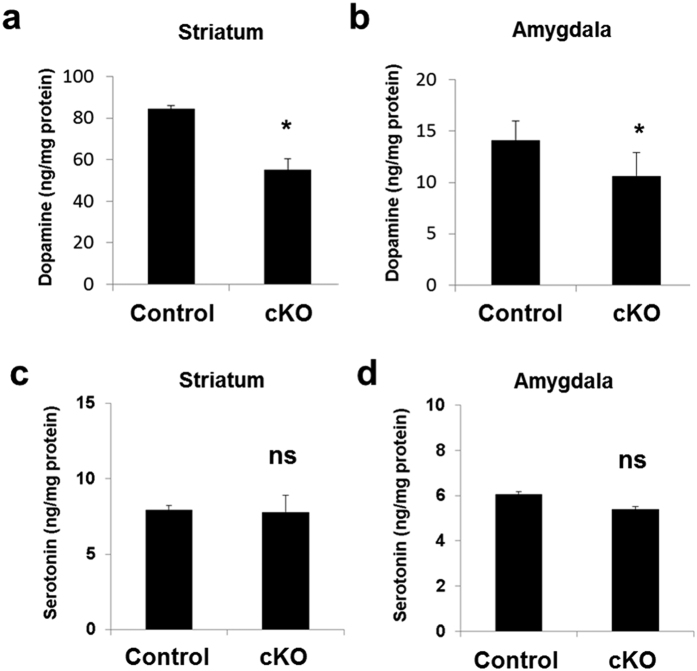
Dopamine content, not serotonin content, is decreased in the striatum and amygdala of *Ndufs4* cKO mice. The concentrations of dopamine (**a,c**) and serotonin (**b,d**) in the striatum (**a,b**) and amygdala (**c,d**) from both *Ndufs4* cKO and control mice were measured. *p < 0.05; n.s. not statistically significant.

We reported that *Ndufs4* cKO mice exhibited age-dependent decline of dopamine content in the striatum^34^. This observation was confirmed using 8-month-old mice (Fig. 2a). There was also a significant decline of dopamine content in the amygdala of *Ndufs4* cKO mice compared to control littermates (Fig. 2b). However, the serotonin content was not reduced in either the striatum (Fig. 2c) or amygdala (Fig. 2d), suggesting that conditional deletion of *Ndufs4* in dopaminergic neurons specifically causes dopamine deficiency.

### Conditional deletion of *Ndufs4* does not impair motor behavior

**Figure 3:**
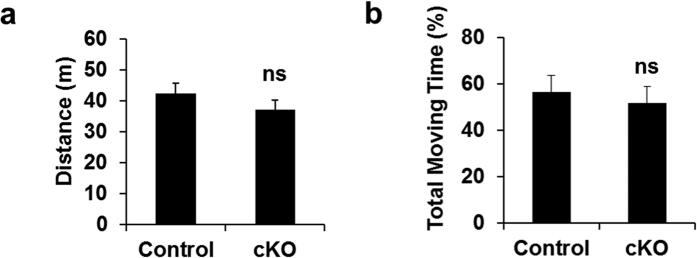
Motor behavior is not affected by conditional *Ndufs4* deletion. Locomotor activity in the open field test was quantified as total distance traveled (**a**), and total moving time (**b**). n.s. not statistically significant.

*Ndufs4* cKO and their littermate controls were subjected to behavioral tests to assess their motor function. *Ndufs4* cKO mice behaved similarly to control mice in the open field test, including the total distance traveled (Fig. 3a) and the total time moving (Fig. 3b). The latency to fall in the rotarod test was also similar (Fig. 3c). These results are consistent with our previous report^34^ and suggest that conditional *Ndufs4* deletion in dopaminergic neurons does not lead to motor deficits.

### *Ndufs4* inactivation in dopaminergic neurons leads to cognitive impairment

**Figure 4:**
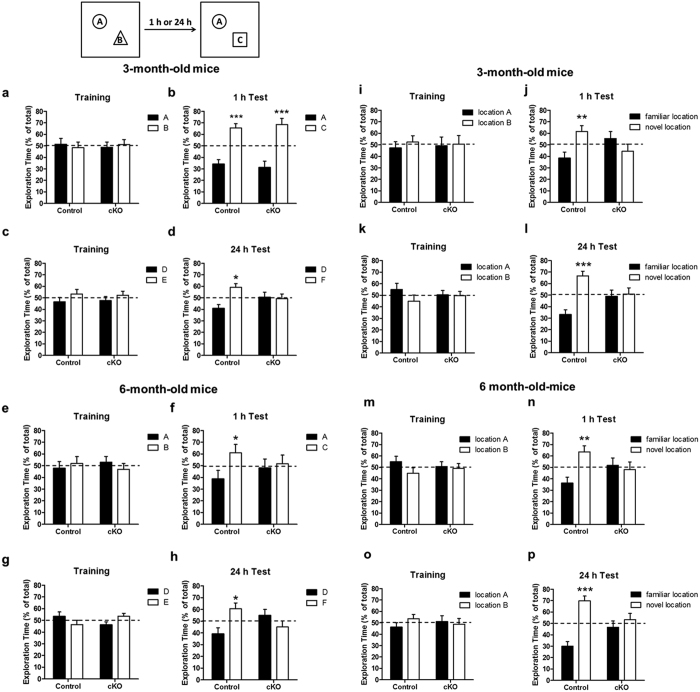
*Ndufs4* cKO mice show progressive memory loss in the novel object recognition assay. (**a–h**) *Ndufs4* cKO and littermate control mice were tested for the novel object recognition assay at 3 months and then again at 6 months of age. The same cohort of mice was subjected to both 1-h and 24-h memory tests on two different days using different sets of objects. (**a,b**)Training and 1 h memory test in 3-month-old mice. (**c,d**) Training and 24-h memory in 3-month-old mice. (**e,f**) Training and 1-h memory in 6-month-old mice. (**g,h**) Training and 24-h memory in 6-month-old mice. n = 7–10 mice/genotype. (**i–p**) *Ndufs4* cKO and littermate control mice were tested for the short-term spatial memory using the novel object location test at 3 months and then again at 6 months of age. Mice were subjected to both 1-h and 24-h memory tests on two different days. **(i,j**) Training and 1-h test using 3-month-old mice. (**k,l**) Training and 24-h test using 3-month-old mice. (**m,n**) Training and 1-h test using 6-month-old mice. (**o,p**) Training and 24-h test using 6-month-old mice. n = 7–10 mice/genotype. *p < 0.05; **p < 0.01; ***p < 0.001.

Impaired cognitive function is one of the common non-motor symptoms associated with Parkinson’s disease. To investigate the effect of conditional reduction of complex I activity on cognitive behavior, control and *Ndufs4* cKO mice were subjected to a novel object recognition test to evaluate both short- and long-term memory. At 3 months of age, both control and *Ndufs4* cKO mice remembered the familiar object “A” equally well 1 h after training and spent significantly more time investigating the novel object “C” during testing, indicating that they had normal short-term memory (Fig. 4a,b). However, unlike control mice, *Ndufs4* cKO did not remember the novel object recognition when the test was performed 24 h after training (Fig. 4c,d). Furthermore, by the age of 6 months, *Ndufs4* cKO mice had lost their 1-h short-term memory as well (Fig. 4e–h). These data suggest that conditional *Ndufs4* deletion in dopaminergic neurons leads to progressive memory loss.

We performed the novel object location test to evaluate spatial learning and memory^47^,^48^. Two identical objects were used in training; one of them was moved to a novel location during tests conducted 1 h or 24 h after training. Control mice, at both 3 and 6 months of age, remembered the object placed at the familiar location 1 h or 24 h after training, spending significantly more time investigating the object in the novel location (Fig. 4i–p). However, the littermate *Ndufs4* cKO mice, at both 3 and 6 months of age, spent an equal amount of time exploring both objects when tested either 1 h or 24 h after training. These results suggest that unlike control mice, the *Ndufs4* cKO mice did not form any short- or long-term spatial memory in this test.

**Figure 5:**
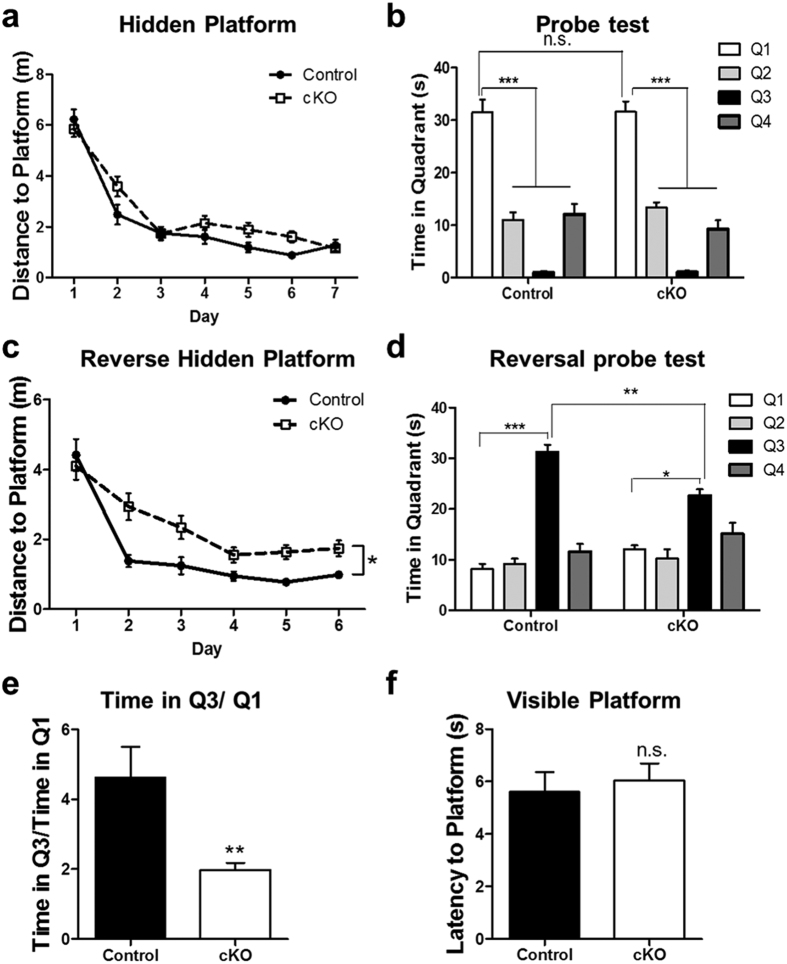
*Ndufs4* cKO mice show impaired spatial learning and memory in the reversal Morris water maze test. (**a**) Target (in quadrant 1) acquisition in the hidden platform, quantified as swim distance to platform, was similar in control and *Ndufs4* cKO mice. (**b**) Both control and *Ndufs4* cKO mice spent more time in the virtual target quadrant (quadrant 1) in the probe test conducted 24 h after training, suggesting that they retained the spatial memory. (**c**) Swim distance to escape in the reverse hidden platform training where the hidden platform was moved to quadrant 3. *Ndufs4* cKO mice swam longer distances to locate the platform, suggesting impaired learning of newer spatial information. (**d**) *Ndufs4* cKO mice spent less time in the virtual target quadrant (quadrant 3) than control mice during the reversal probe test. (**e**) The ratio of time spent in Q3 versus Q1 in the reversal probe test was significantly lower for cKO mice than control mice. (**f**) Control and cKO mice performed equally well in the visible platform test; they showed comparable latency to reach the platform. *p < 0.05; **p < 0.01; ***p < 0.001; n.s. not statistically significant.

Animals were also subjected to the Morris water maze test to further evaluate their spatial learning and memory function (Fig. 5). The *Ndufs4* cKO mice learned to locate the hidden platform as well as control mice over the course of a 7-day training period (Fig. 5a), and had comparable spatial memory as control mice in the probe test conducted 24 h after training (Fig. 5b). However, following a 6-day period of reversal training in which the hidden platform was moved to the opposite location, the *Ndufs4* cKO mice swam longer distances than control mice to find the new platform (Fig. 5c). Furthermore, they spent significantly less time in the new target quadrant than control mice in the probe test after reversal training (Fig. 5d). In addition, although both groups of mice spent more time in the new target quadrant (Q3) than in the old one (Q1) in the reversal probe test, the ratio of time spent in Q3 over that in Q1 was higher for control than for *Ndufs4* cKO mice (Fig. 5e). Two days after the reversal probe test, mice were also subjected to a visible platform test where both groups of mice had similar latencies to reach the platform (Fig. 5f). Thus, the differences observed in the reverse hidden platform training and testing were not caused by differences in vision, ability to swim, or motivation to escape. Reversal training is more demanding and complex than the initial hidden platform training because the mice need to actively forget the old location of the hidden platform and learn to find it in a new location. These results provide further evidence that the *Ndufs4* cKO mice exhibit impaired spatial memory formation.

**Figure 6:**
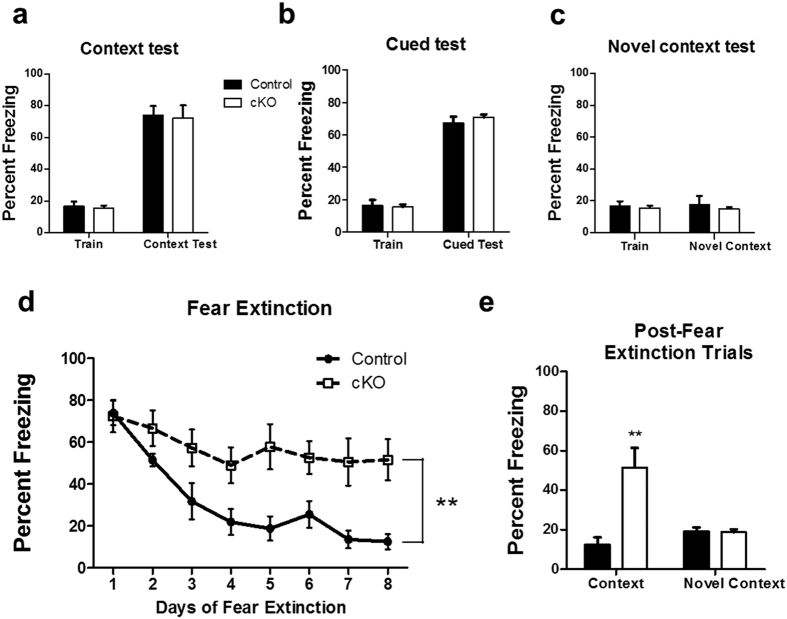
*Ndufs4* cKO mice are deficient in contextual fear memory extinction. (**a**) Control and *Ndufs4* cKO mice have similar levels of contextual fear memory 24 h post-training. (**b**) Control and *Ndufs4* cKO mice showed similar cued-fear response 24 h post-training. (**c**) Neither control nor *Ndufs4* cKO mice froze when placed in a novel context 24 h after training. (**d**) However, fear extinction was impaired in *Ndufs4* cKO mice. (**e**) The freezing response that persisted in *Ndufs4* cKO mice after 8-days of fear extinction training was context specific because the mice did not freeze in a novel context after fear extinction trials. **p < 0.01.

We also performed auditory-cued and contextual fear conditioning as well as contextual fear extinction tests. Auditory cued-fear conditioning requires amygdala function, whereas contextual fear conditioning is hippocampus dependent. Contextual fear extinction is an active form of forgetting in which animals learn to dissociate the context from the foot shock; it is distinct from and may be more complex than contextual fear conditioning^49^. The *Ndufs4* cKO mice manifested normal contextual fear memory (Fig. 6a) and cued-fear memory 24 h after training (Fig. 6b). The contextual memory for both *Ndufs4* cKO and control mice was context specific because the mice did not show freezing when placed in a novel context (Fig. 6c). However, in contrast to control mice, the *Ndufs4* cKO mice did not exhibit fear extinction (Fig. 6d). The contextual memory in the *Ndufs4* cKO mice, which persisted after fear extinction trials, was still context-dependent because the mice did not show freezing when exposed to a novel context after the 8-day fear extinction training (Fig. 6e).

### Loss of *Ndufs4* in dopaminergic neurons induces anxiety-like behavior

**Figure 7:**
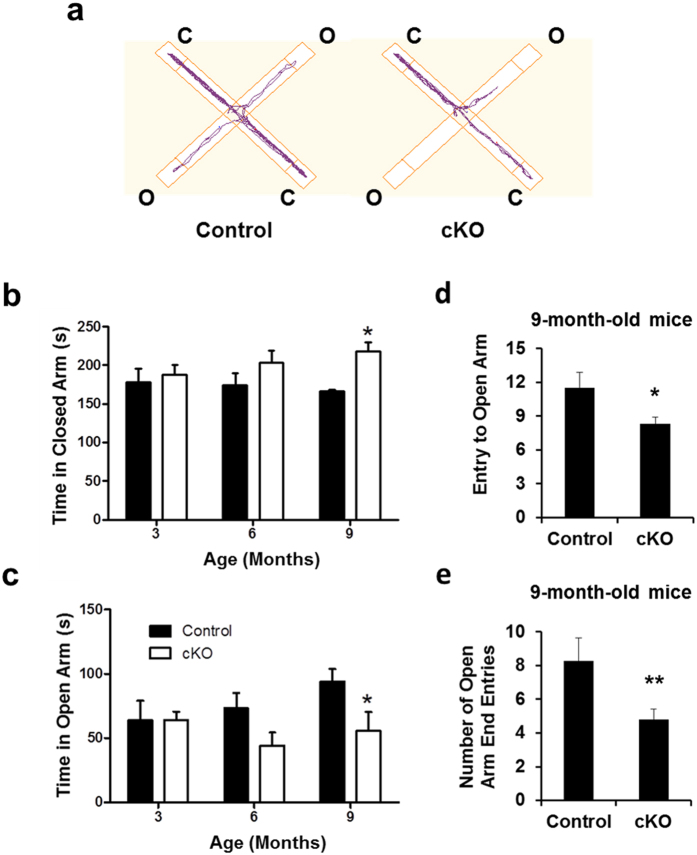
Anxiety-related behavior is enhanced in *Ndufs4* cKO mice in the elevated plus maze test. (**a**) Representative activity traces from a control and an *Ndufs4* cKO mouse in the elevated plus maze test. C, closed arm; O, open arm. (**b**) Time spent in closed arms of an elevated plus maze. (**c**) Time spent in open arms of an elevated plus maze. (**d**) Number of entries to the open arms of an elevated plus maze of 9-month-old mice. (**e**) Number of entries to the open ends of an elevated plus maze of 9-month-old mice. *p < 0.05; **p < 0.01; n.s. not statistically significant.

**Figure 8:**
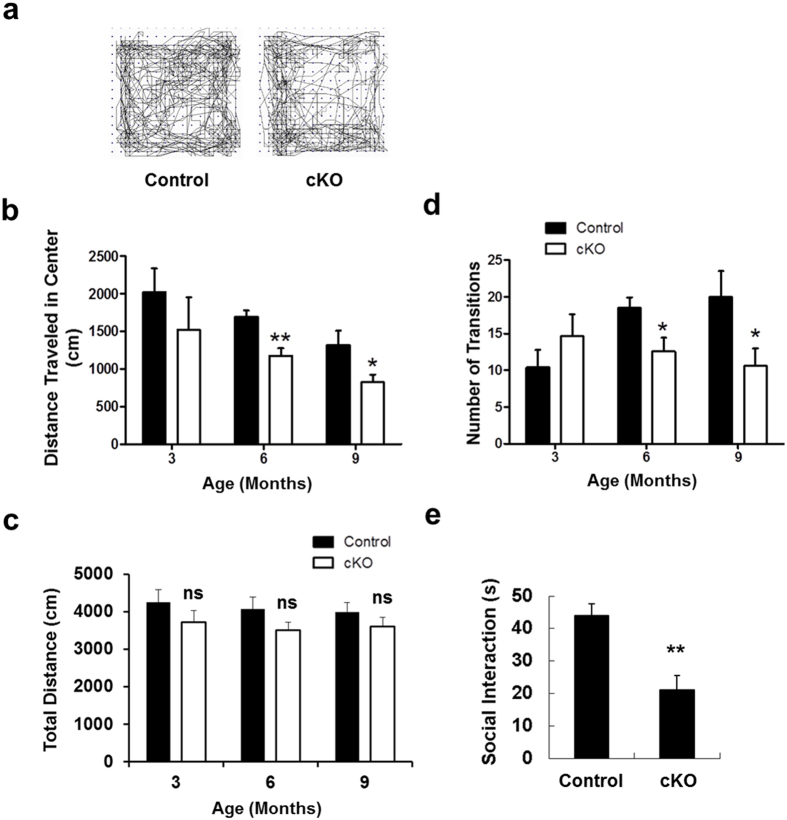
Anxiety-related behavior in the open field. (**a**) Representative activity traces from a control and an *Ndufs4* cKO mouse in the open field test. (**b**) Distance traveled to the center in the open field test. (**c**) Total distance traveled in the entire open field. (**d**) *Ndufs4* cKO mice show increased anxiety in a light/dark exploration test. Data shown are the number of transitions made between the light and dark chamber in a light and dark box. (**e**) Social interaction is reduced in *Ndufs4* cKO mice. Data shown are the time spent in social interactions. *p < 0.05; **p < 0.01; n.s. not statistically significant.

Several behavior tests were performed with 3-, 6-, and 9-month-old mice to investigate the effect of complex I dysfunction on anxiety-like behavior, another common non-motor symptom of Parkinson’s disease. In the elevated-plus maze test, 9-month-old *Ndufs4* cKO mice spent significantly more time in the closed arms (Fig. 7a,b) and significantly less time in the open arms (Fig. 7a,c) compared to control mice. The frequency of entry to the open arms (Fig. 7d) and more specifically, to the open ends (Fig. 7e) was also significantly reduced in 9-month-old *Ndufs4* cKO mice. Starting at 6 months of age, *Ndufs4* cKO mice traveled less distance in the center of the open field (Fig. 8a,b), although their total distance traveled in the entire field was unchanged (Fig. 8c). These mice also made fewer entries to the light box and fewer transitions between the light and dark boxes in the dark/light test starting from 6 months of age (Fig. 8d). In the social interaction test, *Ndufs4* cKO mice showed fewer interactions with other mice (Fig. 8e). Collectively, these data suggest that *Ndufs4* cKO mice exhibit age-dependent, anxiety-like behavior.

## Discussion

Parkinson’s disease patients suffer from both motor and non-motor symptoms. However, the contribution of complex I deficiency to the non-motor symptoms of Parkinson’s disease has not been established and, thus, was the subject of this study. To this end, we utilized a conditional *Ndufs4* knockout mouse we recently generated by crossing *Ndufs4*^loxP/loxP^ mice^31^ with *Slc6a3*^iCre/+^ mice^50^ in which the codon-improved *Cre* recombinase gene (iCre) is expressed under the control of the regulatory elements for the dopamine transporter (DAT) gene, encompassed in a Bacterial Artificial Chromosome (*Slc6a3*). Thus, the *Ndufs4* gene is conditionally deleted in dopamine neurons in our *Ndufs4* cKO mice^34^. In a prior study, Sterky *et al*. crossed the same *Ndufs4*^loxP/loxP^ mice^31^ to mice that express the *cre*-recombinase in heart and skeletal muscle^51^. It was reported that the loss of *Ndufs4* causes only mild reduction of complex I activity in intact mitochondria prepared from heart of these mice^52^. Immortalized mouse embryonic fibroblasts prepared from *Ndufs4*^−/−^ mice showed 35–60% reduction of complex I activity in intact cells^53^. These results led some researchers to argue that *Ndufs4* deletion causes only mild complex I deficiency *in vivo*^52^.

However, we reported that, as a result of *Ndufs4* deletion, complex I-dependent O_2_ consumption is almost completely lost in intact mesencephalic neurons cultured from *Ndufs4*^−/−^ mice^32^. Furthermore, purified DAT^+^ synaptosomes with intact mitochondria from the brains of *Ndufs4* cKO mice showed 84% loss of complex I activity, measured by complex I inhibitor-sensitive oxygen consumption rate using the polarography assay^34^. It is possible that these seemingly different results were obtained by different research groups because different Cre mice were used to delete *Ndufs4*, and different methods were employed to quantify complex I activity in intact mitochondira (ATP produciton vs. O_2_ consumption). Another important difference is the types of tissues used for the assays—heart vs. brain or cultured neurons. It has been documented by an independent group that all investigated tissues of the *Ndufs4*^−/−^ mice show a significant decrease in complex I activity in intact mitochondria compared with the control animals^54^. However, there are significant differences in the residual complex I activities in the different *Ndufs4*^−/−^ tissues; 44% in heart, 26% in brain, and 9% in lung tissues^54^. This 74% decrease of complex I activity in the brain of *Ndufs4*^−/−^ mice is a substantial reduction and consistent with our findings^32^,^34^.

The *Ndufs4* cKO mice did not show loss of dopaminergic neurons in the SNpc or dopaminergic nerve terminals in the striatum and amygdala, and their motor behavior was also normal. These data confirm our previous report that reduction of complex I activity in dopaminergic neurons *per se* is not sufficient to cause dopaminergic neuron degeneration or dopamine-dependent motor deficits^34^. Our data are consistent with other studies suggesting that deficiency of mesencephalic complex I activity does not directly correlate with Parkinson’s disease in mice^52^ or in humans^55^.

Both axons and cell bodies of dopaminergic neurons degenerate during the progression of Parkinson’s disease^56^. At the onset of diagnosis, while only approximately 30% of dopaminergic neuron cell bodies are lost, there is a 50% loss of dopaminergic axon terminals and up to 68~82% decrease of dopamine content in the striatum^57^,^58^,^59^,^60^,^61^. These findings suggest a dying back mechanism; the loss of dopamine precedes the structural degeneration of dopaminergic innervation and cell bodies in the nigrostriatal pathway. Interestingly, mitochondrial complex I deficiency in the *Ndufs4* cKO mice also led to reduced dopamine in the striatum and amygdala without affecting the dopamine nerve terminals in these regions or dopamine neuron cell bodies in the substantia nigra. We do not know the molecular mechanism underlying the reduction of striatal dopamine in the absence of dopamine neuron death in *Ndufs4* cKO mice. The loss is specific to dopamine since serotonin is not affected. Since the reduction of complex I activity leads to reduced ATP synthesis in *Ndufs4* null mice^31^, one possibility is that dopamine synthesis or transport to the nerve terminals in the striatum is inhibited as a result of reduced ATP. Though *Ndufs4* cKO mice have a significant decline (approximately 35% in the striatum) of dopamine content compared to control littermates, this decline was not sufficient to cause motor dysfunction. This is consistent with the literature that much more serve loss of striatal dopamine is required before motor deficits appears in rodents^62^,^63^,^64^,^65^,^66^,^67^,^68^. For example, mice exposed to MPTP can lose up to 50% of dopamine without apparent motor deficits^67^,^68^. Similarly, up to 80% of striatal dopamine is already lost at the onset of Parkinson’s disease diagnosis^69^.

Many Parkinson’s disease patients experience anxiety and depression^35^,^36^,^37^,^70^. Dopamine is important for emotional responses. For example, deletion of VMAT2, which transports cytosolic dopamine into vesicles, increases anxiety in mice^71^. Dopamine signaling during development critically affects aggressive and affective behaviors^72^. Interestingly, dopamine content in the striatum and amygdala of *Ndufs4* cKO mice is lower than that in their controls. The *Ndufs4* cKO mice also showed anxiety-like behaviors in the open field, elevated plus maze, light/dark exploration, and social interaction tests.

Cognitive impairment is also a common non-motor symptom of many Parkinson’s patients^35^,^36^,^37^,^70^. Dopamine is important for learning and memory^73^,^74^. *Ndufs4* cKO mice exhibit impaired learning and memory in several commonly used behavioral tests, including novel object recognition, novel object location, reversal hidden platform Morris water maze, and contextual fear extinction. The defects in hippocampus-dependent spatial memory and extinction of contextual fear exhibited by the *Ndufs4* cKO mice may reflect loss of dopamine and the fact that dopamine receptors in the hippocampus regulate synaptic plasticity and hippocampus-dependent memory^75^,^76^. For example, activation of dopamine receptors in the hippocampus increases long term potentiation (LTP) presumably by stimulation of adenylyl cyclase activity^77^ which is known to be required for hippocampus-dependent memory and LTP^78^.

Disruption of mitochondrial complex I has been implicated in diverse mental disorders including depression^79^,^80^. Down regulation or oxidative damage of complex I subunits and reduced complex I activity were observed in the brains from bipolar disorder patients^81^,^82^. In animal models, complex I inhibitor induced anxiety-like behavior^83^. Because *Ndufs4* cKO mice do not show loss of dopamine neurons or impaired motor functions associated with Parkinson’s disease, our findings that *Ndufs4* cKO mice exhibit cognitive impairment and anxiety may have broader implications for mitochondrial deficiencies in mental disorders in non-Parkinson’s disease patients.

In summary, data presented here provide evidence that reduced activity of mitochondrial complex I in dopaminergic neurons contributes to dopamine loss and non-motor behavior deficits associated with Parkinson’s disease including cognitive impairment and anxiety-like behavior. Furthermore, the *Ndufs4* cKO mice may be a useful model to study the role of mitochondrial deficiencies in mental disorders.

## Methods

### Generation of *Ndufs4* cKO mice

The experimental *Ndufs4* cKO mice (*Ndufs4*^loxP/loxP^:: *Gt(ROSA)Sor*^*fsLacZ/ fsLacZ*^:: *Slc6a3*^iCre/+^) were generated as described^34^. *Ndufs4*^loxP/loxP^::*Gt(ROSA)Sor*^*fsLacZ*/*fsLacZ*^ littermates were used as controls. Mice were housed and maintained in standard laboratory conditions of 12:12 h light:dark cycle. Regular chow and water were provided *ad libitum*. All animal experiments were performed in accordance with the guidelines of the University of Washington and Chonnam National University Institutional Animal Care and Use Committee. All procedures were approved by the University of Washington and Chonnam National University Institutional Animal Care and Use Committee. Male mice were used for behavior experiments.

### Immunohistochemistry and quantification of TH^+^ neurons in the SNpc

Mice were perfused, and the brains were harvested and post-fixed as described^34^,^84^. Brain sections (40 μm) were labeled with either mouse or rabbit antibodies against tyrosine hydroxylase (TH, 1:5,000, Pel-Freez Biologicals, Rogers, AR) in PBS containing 0.1% Triton X-100, 5% BSA, and 5% goat serum. Sections were washed and incubated with Alexa Fluor 488 or 568 goat anti-mouse or anti-rabbit IgG respectively (1:200; Molecular Probes, Eugene, OR) for 1 h at room temperature. Photomicrographs were captured using a fluorescence microscope equipped with a digital camera (Axiovert 200 M, Zeiss, Oberkochen, Germany).

TH^+^ neurons were counted manually, blinded of genotype. Beginning from the first slide of the SNpc section when TH^+^ neurons were visible, all TH^+^ neurons were counted on every fourth slide through the entire SNpc in one brain hemisphere. The estimated total number of TH^+^ neurons in each brain was calculated by multiplying the TH^+^ cell counts by 8 and presented as the total number of TH^+^ neurons in the SNpc of each brain. Six mice from each group were analyzed at 9 months of age.

### TUNEL staining

TdT-mediated dUTP-biotin nick end labeling (TUNEL) staining labels apoptotic cells by detecting 3′-OH ends of broken DNA. DeadEnd™ TUNEL system (Promega, Madison, WI, USA) was used for TUNEL labeling^32^. Briefly, sections were rinsed in 0.1 M PBS 3 times and then incubated in isopropanol solutions, rinsed in 0.1 M PBS, incubated in TUNEL dilution buffer for 2 min, followed by another hour at 37 °C after the addition of terminal deoxynucleotidyl transferase (TdT, 1:3 dilution with TUNEL buffer). Finally the sections were incubated in stop buffer at room temperature for 10 min and rinsed in 0.1 M PBS. Three mice from each group were analyzed at 9 months of age.

### Dopamine and serotonin measurement

The tissue from the striatum or amygdala was dissected, frozen on dry ice, and sent to the Neurochemistry Core Laboratory at Vanderbilt University’s Center for Molecular Neuroscience Research for dopamine quantification. Briefly, the tissues were homogenized in 0.1 M trichloroacetic acid containing 10 mM sodium acetate/0.1 mM EDTA/1 mM isoproterenol (internal standard)/10.5% methanol, pH 3.8, and centrifuged at 10,000 *g* for 20 min. Total dopamine content in the supernatant was quantified by HPLC coupled with electrochemical detection (0.7 V). The HPLC system (Antec Leyden, Zoeterwoude, Netherlands) consisted of a 515 HPLC pump, a 717 plus autosampler, an electrochemical detector (Decade II; Antec Leyden), and an HPLC column (150 × 4.6 mm; Nucleosil C18; Phenomenex, Torrance, CA). The homogenization buffer was used as the mobile phase (0.7 ml/min), and 20 μl of the sample was injected into an HPLC column (3.9 × 300 mm; Nova-Pak C18, Waters Milford, MA). Dopamine or serotonin content was normalized to the protein concentration in each tissue. Four mice from each group were analyzed at 9 months of age.

### Behavior tests

Animals were habituated to the behavior test room for a week before testing. All behavior tests were carried out with male mice between 10:00 am and 6:00 pm and analyzed by experimenters without the knowledge of the genotype and/or treatment.

#### Open field test

The open field test was performed to measure locomotor activity as described^85^. Mice were tested at 3, 6 and 9 months of age (Control n = 8, 14 and 8 respectively; cKO n = 8, 13 and 9 respectively). A TruScan Photo Beam Tracking arena (Coulbourn, Whitehall, PA) was used for the test and TruScan 2.02 software (Coulbourn) was used for data analysis.

#### Rotarod test

The rotarod test was performed as described^84^. Control (n = 16) and cKO (n = 15) mice (9 months old) were placed on the stationary cylinder of the rotarod apparatus (San Diego Instruments, San Diego, CA) and habituated on the apparatus for at least four consecutive trials in which the rod was kept at a constant speed (4 rpm). Once the animals were able to stay on the rod rotating at 4 rpm for at least 60s, they were subjected to the rotarod test. Mice were placed on the rod rotating at an accelerating speed from 4 to 29 rpm in 300s. The time before animals fell off the rod was recorded with a maximum cut-off of 300s. Mice were tested for eight consecutive trials with at least 5-min intervals. The data from the last four trials were averaged as the latency to fall.

#### Novel object recognition test

This assay was done with mice at 3 and 6 months of age (Control n = 7; cKO n = 9 and 10 for 3 and 6 months respectively) as described^48^,^85^. Mice were introduced to 2 objects (A and B, or D and E) during training, and then to one familiar (A or D) and one novel object (C or F) at 1 or 24 h later during the test session. A 5-min session was used for both training and testing. Exploratory activity towards each toy was recorded for both training and testing sessions and analyzed by experimenters blinded to genotype. Significantly more time spent exploring the novel object than the familiar object during testing indicates memory retention for the familiar object.

#### Novel object location memory test

This assay was done with mice at 3 and 6 months of age (Control n = 7 and 9 respectively; cKO n = 9 and 10 respectively) as described^48^. Briefly, a 5-min session was used for both training and testing. Mice were allowed to explore two objects during training. The same two objects were used during testing except that one of them (randomly chosen) was moved to a different location. Exploratory activity towards each object was recorded for both training and testing sessions and analyzed by experimenters blinded to genotype. Increased time spent exploring the object in the novel location during testing indicates retention of spatial memory for the relative locations of the two objects presented during training.

#### Morris water maze assay

This was done as described^85^. Briefly, control (n = 9) and cKO (n = 10) mice (9 months old) were placed in a 1.2-meter-diameter, 25-cm-deep pool of water at room temperature (25 °C). Non-toxic white paint was added to make the water opaque. Three extra-maze cues, different in shape and size were placed on the wall surrounding the water tank. A small escape platform (13 cm x 8 cm) made of clear plexiglass was submerged just below the surface of the water and maintained in a fixed location for the entire training session. A total of 28 trials (4 trials per day for 7 consecutive days) were performed during the training session. Mice were allowed to swim for 40 s to find the platform, or guided to the platform after 40 s of the allotted maximum swim time was reached. Each trial ended after mice were allowed to stay on the escape platform for 15 s. A probe test was performed 24 h after the last training trial in which the escape platform was removed and mice were allowed to swim for 60 s in search of the escape platform. Reversal training ensued 24 h after the probe test for a total of 24 trials (4 trials per day for 6 consecutive days) where the escape platform was placed in the opposite quadrant from the initial training session. A reversal probe test was performed 24 h after the last trial of reversal training. Inter-trial interval was 30 min for all sessions and tests. All data were collected and analyzed using ANYmaze software (San Diego Instruments).

#### Contextual fear conditioning and contextual fear extinction

This was done as described with slight modifications^85^. Briefly, control (n = 8) and cKO (n = 8) mice (9 months old) were placed in a 10″(W) x 10″(D) x 16″(H) square-shaped arena fitted with a metal grid shock floor (Coulbourn). On the day of training, each mouse was placed in the training context (with striped wallpaper) and allowed to freely explore for 2 min. A 90 dB tone, the conditioned stimulus (CS), was then presented for 30s. During the final 2s of tone presentation, a 0.7 mA foot shock, the unconditioned stimulus (US), was delivered. CS and US were delivered automatically using tone generator and shocker controlled by TruScan software (Coulbourn). Mice were then returned to their home cages. Twenty-four hours later, the contextual fear conditioning test was performed in the training room, where mice were placed in the same context arena without any foot shock for 2 min. Two hours after the contextual test, mice were subjected to a cued test. Mice were placed in a novel context (hexagonal shaped arena with clear plastic walls) in a different room and allowed to freely explore for 2 min. The CS (tone) was then presented for 2 min. Two hours after the cued test, mice were subjected to a control test in which they were placed in another novel context (triangular shaped arena with solid grey plastic walls) in a third room for 2 min with no presentation of either tone or foot shock. Freezing behavior was recorded manually every 5s for each of the 2 min assessment periods for the three tests. Freezing behavior was defined as lack of bodily movement with all 4 paws remaining stationary on the floor except normal respiration.

One day following cued and contextual fear conditioning tests, mice were placed in the training context without the foot shock or tone for one extinction trial per day for 8 consecutive days. Each extinction trial lasted a total of 10 min and freezing behavior was recorded during the final 2 min of each trial. One day after the completion of the 8 d fear extinction trials, mice were placed in a novel context (trapezoid shaped arena with solid grey plastic walls) and freezing behavior was recorded for 2 min.

#### Elevated plus maze test

The elevated plus maze test was done as described^86^. The apparatus consisted of two open arms and two enclosed arms arranged in a plus-sign orientation. The arms were elevated 30 inches above the floor, with each arm projecting 12 inches from the center. Mice were tested at 3, 6 and 9 months of age (Control n = 7, 8 and 11 respectively; cKO n = 9, 10 and 11 respectively). Exploratory activities in both open and closed arms were recorded and analyzed using ANYmaze system (Stoelting, Wood Dale, IL) by experimenters blinded to genotype. Because rodents naturally prefer dark and enclosed compartments, a greater willingness to explore the open arms indicates less anxiety while more time spent in the closed arms is indicative of increased anxiety^87^,^88^,^89^.

#### Light/dark box assay

This test was performed as described^90^. A 2-chamber box (10″ width × 5″ depth × 16″ height for each chamber) was used for this test (Light/dark chamber, San Diego Instruments Co). Mice were tested at 3, 6 and 9 months of age (Control n = 7, 8 and 7 respectively; cKO n = 9, 10 and 8 respectively). Mice were habituated in the room for 1 h before the test with the room lights off. During the test, a mouse was placed in the dark chamber and allowed to freely move for 5 min. The time spent in each chamber was scored.

#### Social Interaction Test

The social interaction test was performed as described^91^ in a regular cage. Each of control (n = 8) and cKO (n = 7) mice (9 months old) was placed in the cage for 10 min for habituation. On test day, pairs of unfamiliar mice were placed in the cage for 10 min. Mouse behavior and the time of social interaction was recorded by a computer and the video-tracking system (Stoelting).

### Statistical analysis

All data are expressed as mean ± standard error of the means (s.e.m.). Statistical analysis was performed using Two-way ANOVA with repeated measures to analyze data for the water maze tests and fear extinction assays. One-Way ANOVA was used to analyze the other behavior data. Student’s t-test was used for all the other data, n.s. not significant; *p < 0.05; **p < 0.01; ***p < 0.001.

## Additional Information

**How to cite this article:** Choi, W.-S. *et al*. Conditional deletion of *Ndufs4* in dopaminergic neurons promotes Parkinson’s disease-like non-motor symptoms without loss of dopamine neurons. *Sci. Rep.* **7**, 44989; doi: 10.1038/srep44989 (2017).

**Publisher's note:** Springer Nature remains neutral with regard to jurisdictional claims in published maps and institutional affiliations.
